# Passive ankle exoskeleton assistance selectively modulates triceps surae neural control during balance

**DOI:** 10.1098/rsif.2025.1305

**Published:** 2026-10-14

**Authors:** Raphaël Hamard, François Hug, Taylor J. M. Dick

**Affiliations:** ^1^ Université Bourgogne Europe, INSERM CAPS UMR 1093, Dijon, France; ^2^ School of Biomedical Sciences, The University of Queensland, Brisbane, Queensland, Australia; ^3^ Université Côte d'Azur, LAMHESS, Nice, France; ^4^ Institut Universitaire de France, Paris, Île-de-France, France

**Keywords:** assistive device, electromyography, muscle activation, motor unit, neural drive

## Abstract

Wearable assistive devices have potential to improve human balance, but their effectiveness remains limited by an incomplete understanding of how mechanical assistance influences neuromuscular control. This study aimed to determine the neural adaptations in the control of lower leg muscles in response to passive ankle exoskeleton assistance during standing balance tasks with varying levels of difficulty. Lower limb kinematics and kinetics were recorded to assess changes in joint dynamics and balance performance. Neural drive to the muscles was assessed by decomposing high-density electromyography signals into motor unit spike trains. Ankle exoskeleton assistance, provided by a simple spring-loaded passive device, did not alter or improve balance performance and had minimal effects on lower limb kinematics. However, exoskeleton assistance affected triceps surae neural control, but only in the soleus, by reducing neural drive, while the gastrocnemii showed no detectable changes in neural control. Together, these results demonstrate that passive exoskeleton assistance alters neural control of the triceps surae in a muscle-specific manner during balance. Understanding muscle-specific adaptations can support more effective exoskeleton design and the development of their control strategies informed by myoelectrical signals.

## 1. Introduction

Maintaining balance during standing and walking is a fundamental component of human motor control. It involves controlling the body's centre of mass relative to the base of support to ensure stability. Cognitive, motor and sensory impairments can compromise balance [1,2], substantially reducing independence and quality of life. One approach to restore or augment balance is via the use of wearable assistive devices, such as exoskeletons [3–5]. Devices targeting the ankle joint are particularly prevalent, as ankle moments play a key role in controlling the body's centre of mass [6]. Nevertheless, the widespread adoption of wearable assistive technologies in everyday life remains limited, primarily because we do not yet understand how devices interact with, or respond to, the biological tissues of the user [7]. Optimizing devices requires a comprehensive understanding of their interactions with the neuromusculoskeletal system.

Balance is maintained through the integration of sensory inputs from the somatosensory, vestibular and visual systems [8]. The muscles involved in maintaining balance are controlled through both feed-forward and feedback mechanisms [8,9]. Although wearable assistive devices typically provide torque in an effort to offload postural muscles or to compensate for muscle weakness, assistance may also interfere with these balance control mechanisms. For example, the additional torque provided by a device could influence feed-forward control by modifying the activation patterns of postural muscles. Wearing an exoskeleton providing plantarflexion assistance leads to a reduction in soleus (SOL) activation while simultaneously increasing activation of antagonist tibialis anterior (TA) [4]. Alternatively, external assistance may influence feedback control via altering signals from sensory receptors within skeletal muscle. Specifically, a change in muscle activation alters muscle fibre length and force production [10], which may in turn alter sensory feedback from muscle spindles and Golgi tendon organs. The complex interaction between feed-forward and feedback mechanisms may influence the neural control of balance in unpredictable ways, potentially explaining the inconsistent effects of exoskeleton assistance on balance performance across different studies [3].

Our understanding of neuromechanical adaptation to assistive devices remains limited, in part because the distinct functional roles of synergist muscles are rarely considered when assisting a joint. This is particularly relevant for the individual muscles of the triceps surae, which serve different roles in balance control. SOL contributes primarily to controlling the ankle moment during static balance, whereas gastrocnemius medialis (GM) and gastrocnemius lateralis (GL) have a greater involvement in reactive responses to perturbations [11–13]. Motor unit recruitment also varies across the triceps surae muscles during balance, which depends in part on the direction of body lean [14–17]. These differences may reflect not only muscle-specific mechanical contributions to ankle torque [18] but also variations in muscle spindle density [19], or intrinsic motor unit properties [12,20]. Considering the behaviour of the individual muscles within the triceps surae is essential for a comprehensive understanding of the neural adaptation to wearable assistive devices. In addition, a muscle-specific approach could inform the development of assistance strategies based on neural signals [21] by identifying the most relevant muscle(s) for effective control interfaces.

The aim of this study was to investigate the neural adaptations of individual lower leg muscles to passive ankle exoskeleton assistance during standing balance tasks with varying levels of postural control difficulty. Electromyography (EMG) signals were recorded using high-density surface electrode arrays placed over the SOL, GM, GL and TA muscles. These signals were decomposed into individual motor unit spiking activity. A major advantage of high-density EMG and its associated decomposition over traditional interference EMG is that motor unit activity provides more direct insight into the neural drive to the muscles, while being insensitive to crosstalk—a common limitation when recording interference EMG signals from the ankle muscles [22]. We expected a reduction in the overall triceps surae neural drive during balance tasks with exoskeleton assistance compared with unassisted balance, but we hypothesized that these adaptations would be muscle-specific.

## 2. Material and methods

### 2.1. Participants

A total of 20 physically active males (means ± s.d.; age: 25.9 ± 8.6 years, height: 179 ± 9 cm, body mass: 79.4 ± 13.7 kg) provided informed written consent to participate in the study, which was previously approved by the institutional ethics review committee at The University of Queensland (no. 2019/HE002595). Of note, only male participants were recruited to maximize the yield of motor units, as EMG decomposition approaches often fail to identify a sufficient number of motor units in female participants for certain muscles, such as the GL [23–25]. Participants had no pre-existing neuromuscular disease or history of lower leg injury in the previous 12 months.

### 2.2. Experimental design

The experimental protocol consisted of four balance tasks of varying difficulty: (i) standing with eyes open, (ii) standing with eyes closed, (iii) standing on a foam surface with eyes closed, and (iv) standing only on a single (dominant) leg with eyes open. Each task was repeated in two different conditions: without the passive ankle exoskeleton and with passive ankle exoskeleton assistance. Participants performed the two conditions in a randomized order, and the four tasks were also randomized within each condition. Each trial lasted 60 s, and for each task except the dominant leg standing, a 22 cm distance between the feet was maintained for the balance task. During each trial, participants stood on an instrumented treadmill (FIT5, Bertec Inc., Columbus, OH, USA) that comprised two force plates to record ground reaction force and centre of pressure (CoP) of each leg. In addition, we recorded surface EMG signals from GM, GL, SOL and TA muscles of the dominant leg using high-density grids of 64 electrodes (EMG-Quattrocento, 400-channel EMG amplifier; OT Bioelettronica, Italy). We also recorded the three-dimensional positions of reflective markers placed on the pelvis, lower limbs and assistive device using a three-dimensional motion capture system (120 Hz, Miqus, Qualisys AB, Göteborg, Sweden).

### 2.3. Passive ankle exoskeleton device

Participants wore bilateral passive exoskeletons that provided plantarflexion torque (figure 1). The assistive device was a custom-built passive ankle exoskeleton previously described by Williamson *et al.* [26]. It consisted of shoe and shank attachments connected by two aluminium side bars. A steel coil spring (stiffness: 13.1 kN m⁻¹) was mounted posteriorly, in parallel with the triceps surae muscles, to provide plantarflexion assistance. The exoskeleton provided a plantarflexion moment about the ankle joint with a moment arm of 0.13 m and had a total unilateral mass of 0.5 kg. Considering the spring stiffness and the moment arm of the device, the projected ankle stiffness was 221 Nm rad⁻¹, which represents approximately 75% of the biological ankle stiffness measured during quiet standing (298 Nm rad⁻¹; [27]).

**Figure 1.**
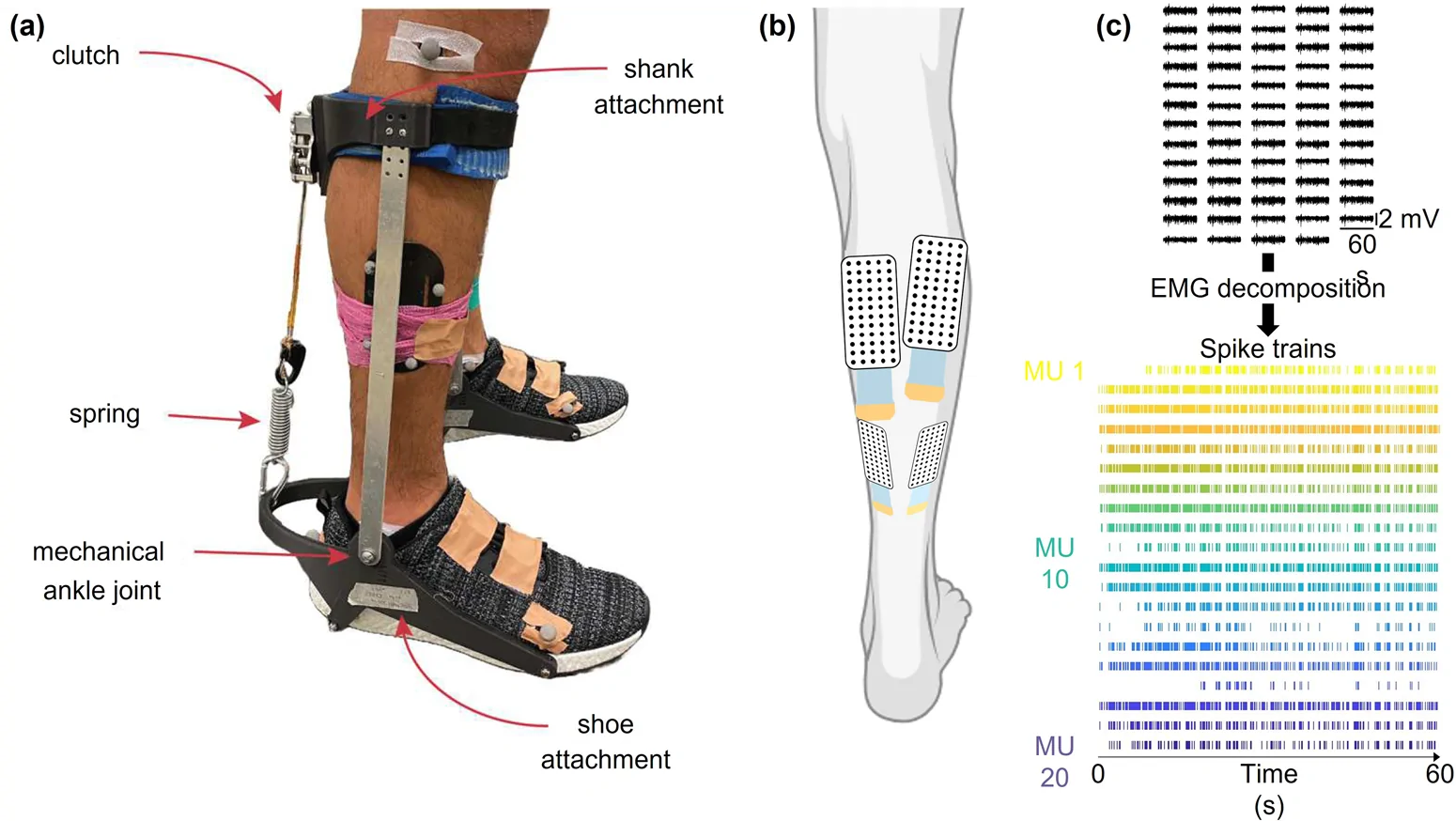
Exoskeleton device and EMG set-up. (a) The passive ankle exoskeleton used in this study, adapted from Williamson *et al.* [26]. (b) Representation of the high-density electrode location from a posterior view. For the GM and GL, one large grid of 64 electrodes was placed on each muscle. For the SOL, two small grids of 64 electrodes were placed on each medial and lateral part of the muscle. Note that grids on TA are not depicted. (c) Representative EMG signal of the GM muscle on the top panel and the resulting motor unit activity after the decomposition on the bottom panel.

The rigid component of the exoskeleton was adapted to the participant's morphology by adjusting the height of the shank attachment and by using foam and straps to ensure skin–device contact at the proximal part of the lower leg. To ensure effective assistance during standing balance tasks, the device's slack length was adjusted to engage when the heel was approximately 2 cm elevated from the ground surface such that the device provided assistance in neutral standing.

### 2.4. Centre of pressure

The three-dimensional ground reaction forces were recorded using two force plates at a sampling rate of 1200 Hz. For the bilateral standing tasks, we ensured that each foot was on a separate force plate. We filtered the ground reaction forces using a second-order Butterworth filter with a 6 Hz cut-off frequency, applied in a forward–reverse manner using the filtfilt function in MATLAB, resulting in zero-phase filtering. We used the ground reaction force from each force plate to calculate the CoP. Then, the whole-body CoP was computed by averaging the CoP coordinates from the two force plates, weighted by the vertical ground reaction force recorded on each plate. Finally, we calculated the total CoP path length displacement for each trial as a proxy for balance performance, consistent with previous studies [28]. CoP data were analysed over the middle 50 s of each trial, excluding the first and last 5 s.

### 2.5. Lower limb kinematics

An eight-camera motion capture system recorded the three-dimensional positions of 36 reflective markers placed bilaterally on bony landmarks of the lower limbs and pelvis (i.e. anterior superior iliac spine; posterior superior iliac spine; greater trochanter; medial and lateral femoral condyles; medial and lateral malleolus; calcaneus; first metatarsal head; fifth metatarsal head; cluster of four markers on the thigh; cluster of four markers on the shank). The marker set also comprised four rigid body clusters of four markers each placed bilaterally on the thighs and shanks consistently with previous studies [26,29]. We filtered the three-dimensional marker trajectories using a zero-phase second-order low-pass Butterworth filter with a 10 Hz cut-off frequency. For each participant, we scaled a musculoskeletal model (OpenSim v. 4.4; [30,31] based on a static standing trial. We used the scaled model and the three-dimensional trajectories of the markers in inverse kinematics analyses to determine the time-varying joint angles of the ankle, knee and hip joints during each trial. From these time-varying signals, we extracted mean flexion angle and the range of motion of the ankle, knee and hip over the middle 50 s of each trial.

### 2.6. High-density surface electromyography

A multi-channel high-density EMG acquisition system was used to collect surface EMG signals from the GM, GL, SOL and TA using two-dimensional adhesive grids of 64 electrodes. For the GM and GL, one large grid was placed on each muscle (13 × 5 electrodes with one electrode absent on a corner, gold-coated, interelectrode distance: 8 mm; GR08MM1305, OT Bioelettronica, Italy). For the SOL and TA, two small grids were placed on each muscle (13 × 5 electrodes with one electrode absent on a corner, gold-coated, inter-electrode distance: 4 mm; GR04MM1305, OT Bioelettronica, Italy). On the SOL, the electrodes were placed on the postero-medial and the postero-lateral regions of the muscle. On the TA, the electrodes were placed on the proximal and distal regions of the muscle. Before placing the electrodes on the muscles, we shaved, cleansed and abraded the skin with abrasive gel (Nuprep, Weaver and Company, Aurora, CO, USA) to reduce the skin–electrode impedance. We used a reference electrode over the patella of the dominant leg and a ground electrode around the ankle of the non-dominant leg. We secured the electrodes with elastic bandages to ensure skin–electrode contact throughout the entire experiment. The monopolar signals were band-pass filtered (10–500 Hz) by the EMG-Quattrocento system and digitized at a sampling rate of 2048 Hz.

EMG amplitude was calculated from the recorded interference EMG signals. Before analysis, each signal was visually inspected over the entire duration of the trial. Channels were excluded if they contained visible movement or electrical artefacts, or if the signal-to-noise ratio was low. Differential signals were then computed by subtracting signals from adjacent electrodes in the proximo-distal direction, yielding 59 differential signals per grid. Consistent with the CoP data, we calculated the root mean square (RMS) over the central 50 s of each trial. EMG RMS values were averaged over the whole grid to obtain a single representative value per grid. For the SOL and TA, we averaged the values from the two grids to obtain one representative value for each muscle.

To gain more direct insight into the neural drive to the muscles, the EMG signals were decomposed into motor unit spiking activity. To this end, we used the MUedit software [32], which is based on convolutive blind source separation. The decomposition was performed on approximately 20 s of data from the middle of each trial, using only the channels free of noise or artefacts. The resulting spike trains were then manually edited according to established procedures [32,33], with the aim of removing the false positives selected by the automatic decomposition and adding the false negatives that were not selected by the automatic decomposition. The motor unit identification filter derived from this processing was then reapplied to the whole trial to assess the motor unit activity on the whole trial.

To enable direct comparison across control and assisted balance conditions, we tracked motor units that were identified in both conditions. To do this, we used the spatio-temporal properties of the action potential waveforms over the grid [34–36]. First, the motor unit action potential waveform was identified using the spike-triggered averaging technique over each of the 59 electrodes (differential mode). The motor unit action potentials from the 59 channels were concatenated and cross-correlated across conditions. The pairs of motor units with a coefficient of correlation greater than 0.75 were considered to represent the same motor unit. Each pair was then visually inspected to verify the consistency of the action potential waveform. Specifically, we checked that the motor unit action potential shapes were similar between the two motor units over the entire grid and removed the pair of motor units that did not meet this criterion to ensure the quality of the matching. For these successfully tracked motor units, we calculated the mean discharge rate for each trial by averaging the instantaneous discharge rate over the middle 50 s of the trial, excluding the first and last 5 s.

### 2.7. Statistical analysis

All statistical analyses were performed with R (v. 4.4.1, Vienna, Austria). We used linear mixed effects models (lme4 package; [37]) to compare the CoP path length displacement, mean joint angles and global surface EMG between conditions (i.e. control and exoskeleton) and between tasks (i.e. standing eyes open, standing eyes closed, standing on a foam surface eyes closed and single-leg standing). We used a within-participant design with condition and task specified as fixed factors and participant specified as a random factor. Before performing the statistical analyses, model assumptions were assessed by visual inspection of diagnostic plots. Specifically, normality of residuals was confirmed using both quantile–quantile (Q–Q) plots and histograms of the model residuals accounting for both the fixed and random effects. Homoscedasticity was confirmed by examining residual versus fitted value plots. We used the emmeans function to perform Tukey's post hoc pairwise comparison when a main effect or an interaction effect was identified. For the surface EMG, we ran a linear mixed effects model for each muscle. We also used linear mixed effects models to compare the discharge rate of tracked motor units between conditions (i.e. control and exoskeleton), but as the motor units tracked can be different between tasks, we ran these linear mixed effects models within each task. Therefore, for the motor unit discharge rate, we ran a linear mixed effects model for each muscle and each task. For all tests, we used an adjustment method based on Bonferroni correction and the level of significance was set at *p* < 0.05.

## 3. Results

### 3.1. Balance performance

Balance performance varied between tasks but not with assistance. Analysis of CoP path length displacement revealed a significant main effect of task (*p* < 0.001), but no main effect of condition (*p* = 0.729) and no condition × task interaction (*p* = 0.878, figure 2). Pairwise comparisons for the task effect were all significant (all *p* ≤ 0.027), indicating a progressive increase in CoP path length with increasing difficulty of the balance task, i.e. from standing eyes open, standing eyes closed, standing on foam eyes closed to single-leg standing.

**Figure 2.**
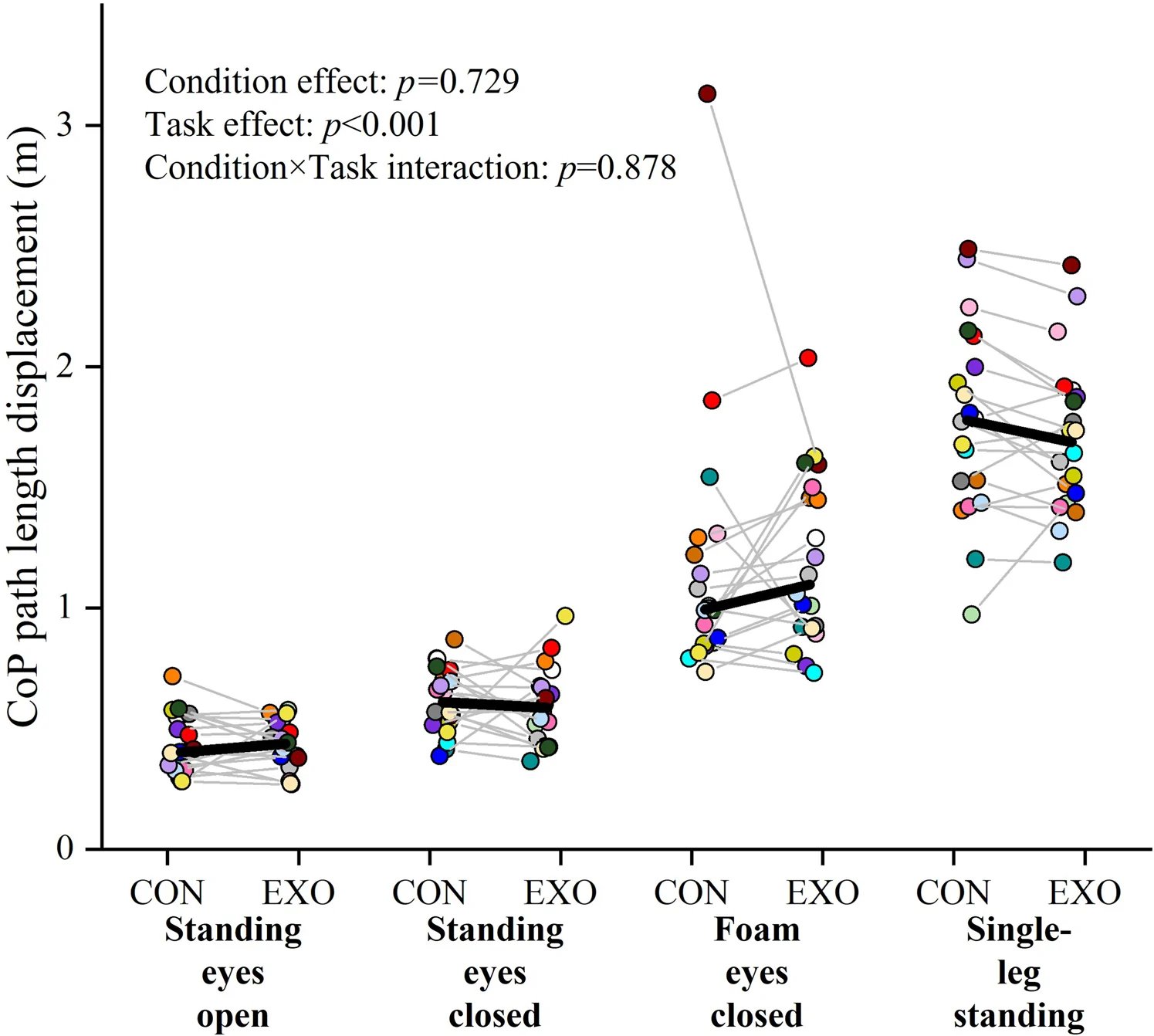
Balance performance varied between tasks but not with assistance. CoP path length displacement measured during control (CON) and exoskeleton (EXO) conditions. Each colour represents an individual participant, with the group mean shown in black. Thin grey lines connect the two conditions for each participant. Pairwise comparisons for the task effect were all statistically significant.

### 3.2. Kinematics

Lower limb kinematics were minimally affected by exoskeleton assistance. Analysis of the mean ankle flexion angle revealed a significant main effect of task (*p* < 0.001) and a significant condition × task interaction (*p* = 0.022), but no significant effect of condition (*p* = 0.831; figure 3). Post hoc analysis showed that the ankle was, on average, 2.4° ± 2.5° more dorsiflexed during single-leg standing compared with the three other tasks, but only in the control condition (all *p* < 0.001), with no between-task difference in the exoskeleton condition (all *p* ≥ 0.102). Analysis of mean knee flexion angle revealed a significant main effect of condition (*p* < 0.001) and task (*p* < 0.001), but no significant condition × task interaction (*p* = 0.598). Specifically, the knee was slightly more extended by 1.5° ± 2.2° during the exoskeleton condition compared with the control condition. The pairwise comparisons for the task effect showed that knee flexion was, on average, 3.5° ± 3.9° greater during single-leg standing compared with the three other tasks (all *p* < 0.001), with no differences between the other tasks. Analysis of mean hip flexion revealed a significant main effect of task (*p* = 0.009), but no main effect of condition (*p* = 0.276) and no condition × task interaction (*p* = 0.912). The pairwise comparisons for the task effect showed that the hip flexion was, on average, 1.9° ± 5.4° lower during single-leg standing compared with the three other tasks (all *p* < 0.001), with no difference between other tasks (all *p* ≥ 0.780).

**Figure 3.**
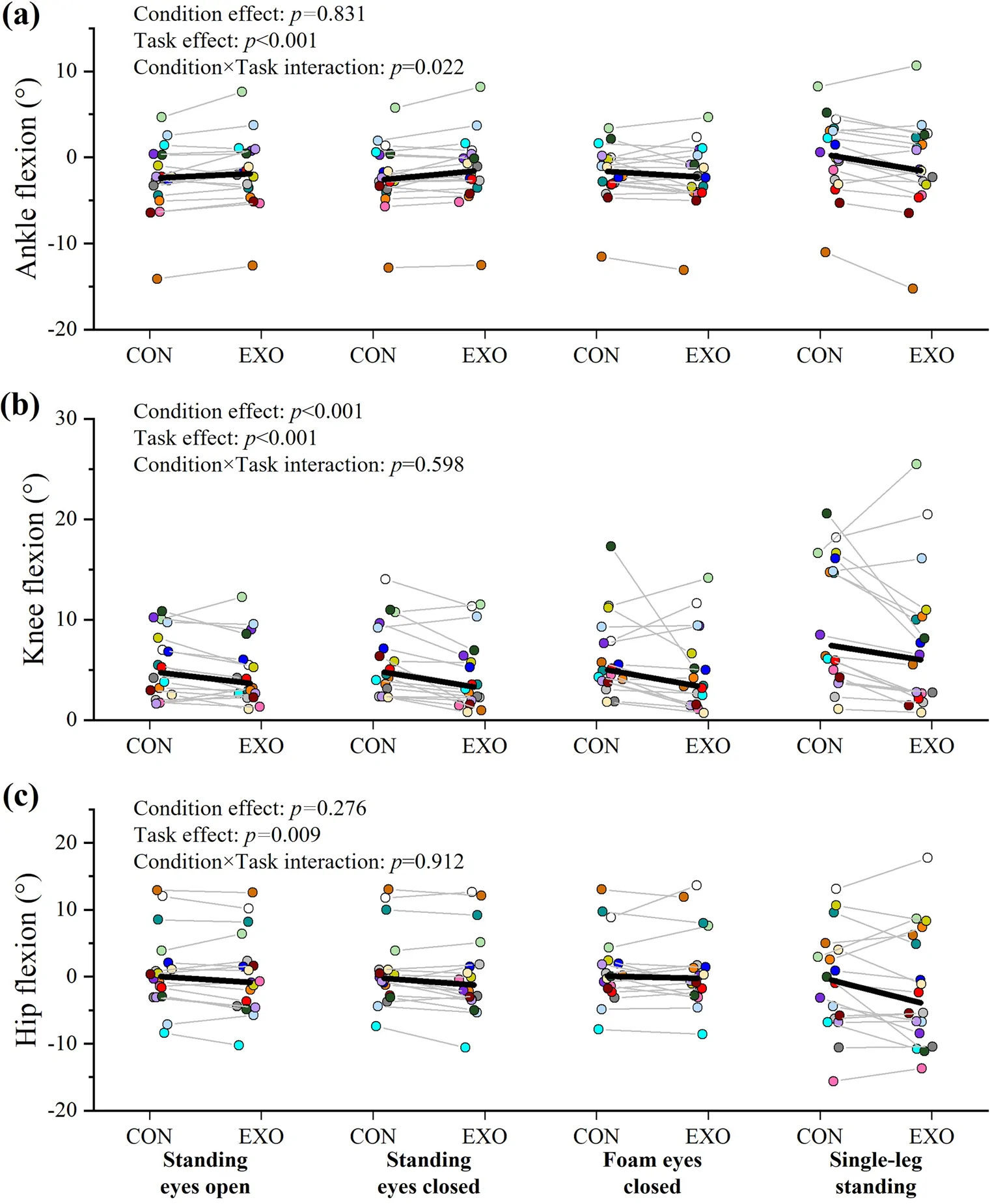
Lower limb kinematics were minimally affected by exoskeleton assistance. (a) Mean ankle flexion, (b) knee flexion and (c) hip flexion angles measured during control (CON) and exoskeleton (EXO) conditions. Each colour represents an individual participant, with the group mean difference shown in black. Thin grey lines connect the two conditions for each participant.

### 3.3. Interference electromyography

The amplitude of the interference EMG signals (EMG RMS) was influenced by exoskeleton assistance for SOL but not for GM, GL or TA. Linear mixed effect models applied independently to the GM, GL and TA revealed a significant main effect of task (all *p* < 0.001), with no main effect of condition (all *p* ≥ 0.428) and no condition × task interaction (all *p* ≥ 0.711, figure 4). The pairwise comparisons for the task effect indicated that GM, GL and TA EMG RMS were higher during single-leg standing compared with the three other tasks (all *p* < 0.001), with no significant differences among standing eyes open, standing eyes closed and standing on foam with eyes closed (all *p* ≥ 0.099). For SOL, EMG RMS showed a significant main effect of both condition (*p* < 0.001) and task (*p* < 0.001), with no significant condition × task interaction (*p* = 0.110). Specifically, SOL EMG RMS was consistently lower in the exoskeleton condition compared with the control condition, with an average reduction of 19 ± 16%. Regarding the task effect, pairwise comparisons revealed that the SOL RMS EMG was, on average 80 ± 34% higher during the single-leg standing compared with the three other tasks (all *p* < 0.001), with no significant differences among standing eyes open, standing eyes closed and standing on foam with eyes closed (all *p* ≥ 0.379). Similar results were obtained when considering the SOL medial and lateral electrode grids separately.

**Figure 4.**
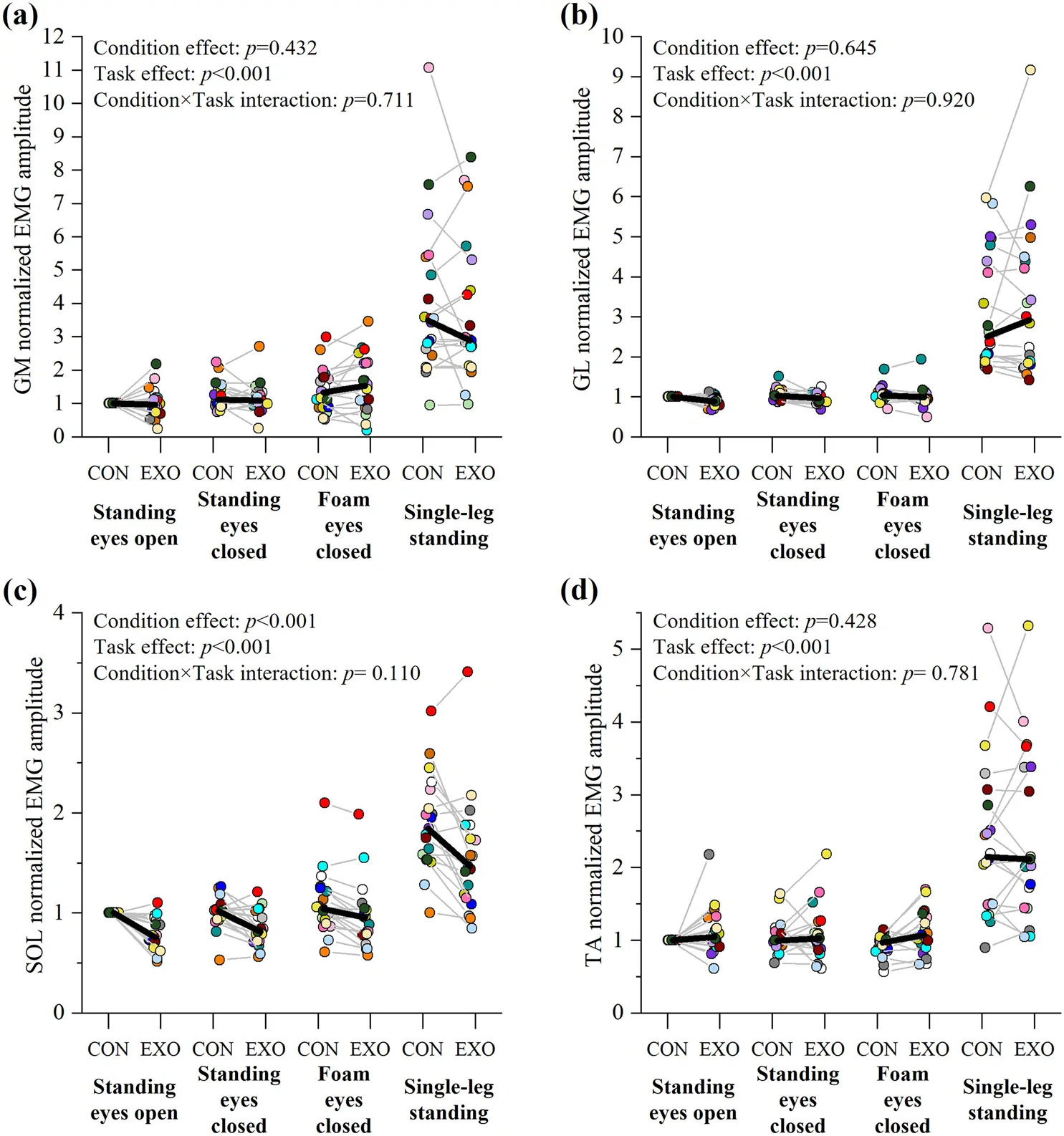
Global muscle activation was influenced by exoskeleton assistance for SOL but not for GM, GL and TA. Normalized surface EMG amplitude measured during control (CON) and exoskeleton (EXO) conditions for the (a) GM, (b) GL, (c) SOL and (d) TA muscles. EMG RMS values were normalized to those obtained in the control condition during the standing eyes open task. Each colour represents an individual participant, with the group mean difference shown in black. Thin grey lines connect the two conditions for each participant.

### 3.4. Motor unit discharge rate

We identified the spiking activity of individual motor units through EMG decomposition and were able to identify a substantial number of motor units only for GM and SOL (table 1). Of note, the lower number of motor units identified in GL (13 motor units) and TA (eight motor units) probably reflects the low activation level of these muscles, consistent with previous findings for GL [12,24], rather than a limitation of the decomposition approach itself.

**Table 1.** Number of identified and tracked motor units across tasks and conditions.

	standing eyes open				standing eyes closed				standing on foam eyes closed				single-leg standing			
	no. of identified MUs		tracked MUs		no. of identified MUs		tracked MUs		no. of identified MUs		tracked MUs		no. of identified MUs		tracked MUs	
	CON	EXO	no.	%	CON	EXO	no.	%	CON	EXO	no.	%	CON	EXO	no.	%
GM	139	120	55	46	167	166	75	45	214	182	83	46	134	132	59	45
SOL	106	71	13	18	121	87	30	34	85	79	44	56	38	30	9	30

*Notes:* The percentages (%) were calculated based on the number of motor units tracked across the two conditions relative to the total number of motor units identified in the exoskeleton condition. CON, control condition; EXO, exoskeleton condition; GM, gastrocnemius medialis; MUs, motor units; SOL, soleus.

All subsequent results were based solely on the motor units that were successfully tracked across the two conditions (i.e. control and exoskeleton). Although the yield was lower than for the untracked motor units, this approach ensured that comparisons across conditions were not confounded by differences in the intrinsic properties of the identified motor units. Therefore, any difference in discharge rates across conditions can be interpreted as a difference in neural drive. For GM motor units, discharge rates did not significantly differ between conditions for any of the tasks (standing eyes open (*p* = 0.868), standing eyes closed (*p* = 0.542), standing on foam eyes closed (*p* = 0.059) and single-leg standing (*p* = 0.181), figure 5). By contrast, for SOL, motor unit discharge rates were significantly lower in the exoskeleton condition compared with the control condition during standing eyes open (*p* = 0.013), standing eyes closed (*p* = 0.017) and standing on foam eyes closed (*p* < 0.001). No significant difference in motor unit discharge rate was observed between conditions during single-leg standing (*p* = 0.113).

**Figure 5.**
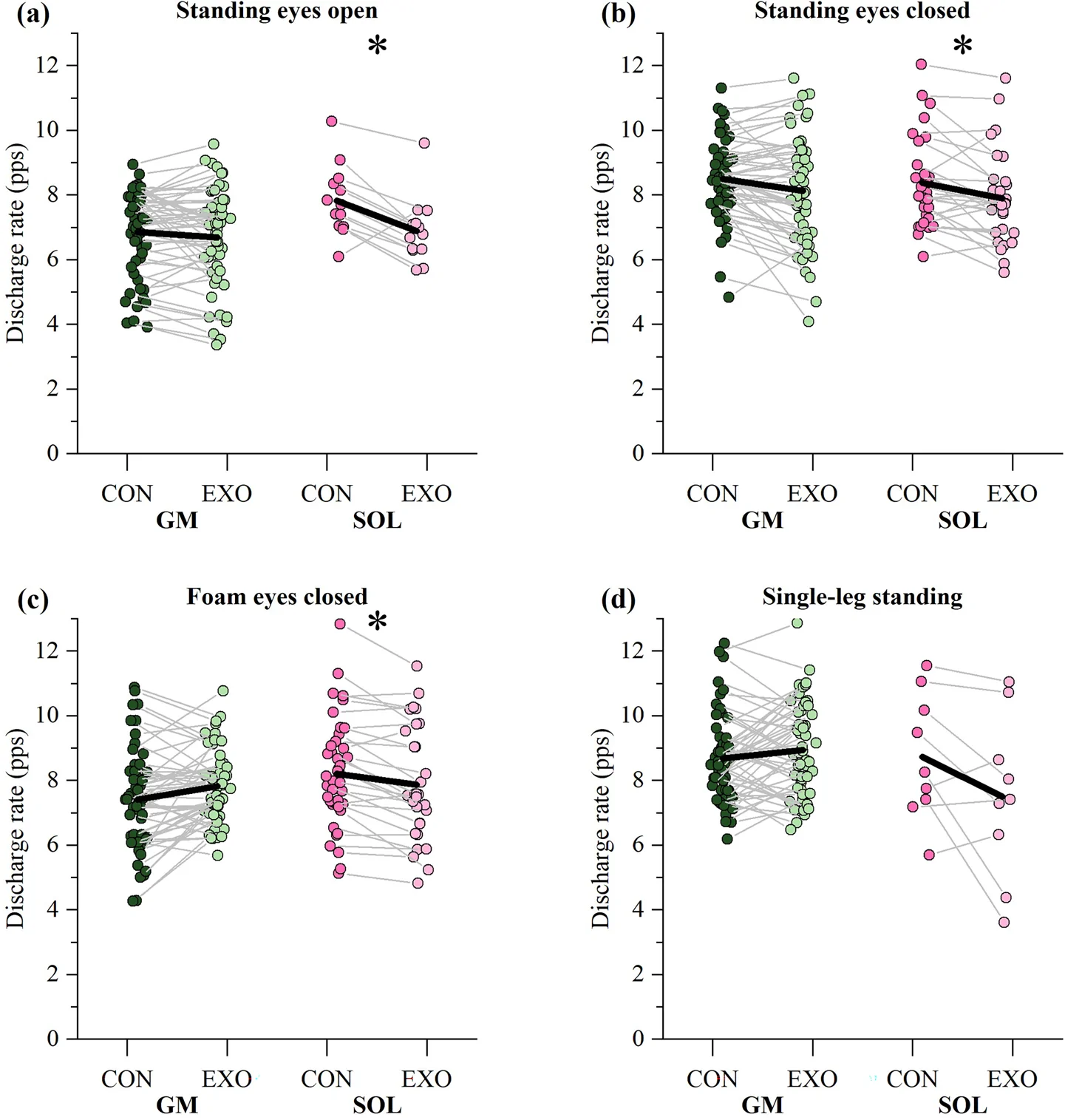
Exoskeleton assistance influenced neural drive to the SOL, but not the GM. Discharge rate of the GM and SOL tracked motor units during control (CON) and exoskeleton (EXO) conditions for (a) standing eyes open, (b) standing eyes closed, (c) foam eyes closed and (d) single-leg standing. The group mean difference is shown in black. Thin grey lines connect each individual motor unit across the two conditions. The asterisks represent a significant difference between conditions.

## 4. Discussion

Wearable assistive devices can improve balance, but their effectiveness is restricted by our limited knowledge of how mechanical support affects neuromuscular control. This study aimed to determine the muscle-specific neural adaptations to passive ankle exoskeleton assistance during balance tasks with varying levels of difficulty. Our main findings were twofold: (i) external assistance affected neural control only in the largest monoarticular muscle, SOL, through a reduction in neural drive, and (ii) passive exoskeleton assistance did not influence static balance performance as quantified by the CoP path length displacement, with minimal effects on lower limb kinematics. Overall, these results provide evidence that passive ankle exoskeleton assistance influences neural control of triceps surae in a muscle-specific manner.

Although the spring-loaded passive device provided assistance at the joint level, our results revealed that its effect differed across the plantar flexor muscles, regardless of task difficulty. We observed that assistance primarily offloaded the neuromechanical demands of SOL by reducing its neural drive. Importantly, this effect was observed across both interference EMG and motor unit analyses. Since we were able to track the same motor units across conditions, a change in discharge rate can be directly interpreted as a change in net synaptic input, reinforcing the robustness of this finding. This muscle-specific exoskeleton effect is perhaps not surprising, given the functional role of SOL during standing balance. The external assistance provided by the device is designed to offload the relatively continuous activation of SOL during static standing balance [11–13]. By contrast, GM and GL, which are more engaged in reactive postural responses characterized by bursts of activation [11,13], were minimally affected by the device. As the present study focused on static balance tasks, further research is required to determine whether a similar device might influence a participant's ability to generate rapid reactive responses during perturbations to standing balance.

The muscle-specific adaptation to the exoskeleton could be relevant for the design and control of adaptive exoskeletons. Several powered exoskeletons have been developed to deliver assistance based on myoelectric signals from lower limb muscles [3,38–40]. In most of these studies, the myoelectric signal from only one muscle, either GM, SOL or TA, was used as an input to the controller of the device. Our results suggest that the muscle selected for deriving the myoelectric control signal may substantially influence the assistance delivered to its user. This highlights the potential importance of integrating signals from multiple muscles, or carefully selecting the most relevant muscle based on the user, the task or the intention of the device.

The passive ankle exoskeleton used in this study reduced overall plantarflexion muscle activation while maintaining similar whole-body and joint dynamics. Of note, the absence of changes in balance performance induced by the device was expected for two main reasons. First, participants were healthy individuals with no known balance deficits. Second, the level of assistance was relatively moderate. The reduction in total SOL active volume associated with wearing the exoskeleton may result in an increased metabolic efficiency of standing balance [41,42]. Although the influence of a 10% reduction in SOL neural drive on the overall triceps surae efficiency may appear modest, it is important to consider that the SOL volume contributes more than half of the triceps surae volume [43–45]. Moreover, humans spend several hours per day standing [46], and such reduction may have a substantial metabolic impact over longer timescales. Our results are compatible with assistance promoting a reduction in metabolic cost; however, our analyses are limited to the triceps surae muscle group and other leg muscles may counteract these effects. Further studies using measures of metabolic cost (i.e. indirect calorimetry) and EMG measures from a greater number of lower limb muscles would provide further insights.

The reduction in SOL neural drive induced by the assistive device may result in neuromechanical adjustments that influence both feed-forward and feedback components of balance control. Ankle angle remained unchanged, whereas the neural drive to the SOL decreased during assistance. This leads to longer SOL muscle fibres, consistent with observations made during walking with an ankle exoskeleton [47]. However, longer SOL fibres should increase feedback from muscle spindles and thereby increase the neural drive to SOL. This prediction is inconsistent with our results, which show a decrease rather than an increase in SOL neural drive. It is therefore more likely that the observed changes arise from a reduction in feed-forward balance control during the exoskeleton condition. Experiments using more direct measures of muscle sensory function (e.g. microneurography or H-reflex) could potentially reveal the influence of assistance on both feed-forward and feedback control.

The analysis of motor unit behaviour provides important insights into the effects of exoskeleton assistance on the neural control of muscles; however, the number of identified motor units was limited in some muscles and conditions. Only nine motor units were identified in SOL during single-leg standing, which may explain why no statistically significant difference between conditions was detected, despite a substantial difference in EMG RMS. In addition, too few motor units were identified in GL and TA to allow meaningful analyses, probably because these muscles were largely inactive under most of the tested conditions, as previously reported during standing with eyes open [12,48]. In the single-leg standing task, GL and TA EMG RMS increased compared with the other tasks, but despite this, we were unable to identify a sufficient number of active motor units. This is probably related to the intermittent muscle activation during this task, which limits the effectiveness of the motor unit decomposition process [49,50]. Further studies using experimental balance tasks that induce a more stable muscle activation of the GL and TA [11,17] could be performed to confirm whether assistance influences neural drive to TA and GL. Of note, the requirement of relatively stable contractions to decompose high-density EMG signals into motor unit activity currently limits the possibility of applying this technique to locomotion. Future developments in decomposition algorithms will probably make it possible to extend this approach to more dynamic tasks, such as walking.

This study contains some limitations. First, we did not measure the assistive forces provided by the exoskeleton. However, we set up the device's slack length on a participant-specific basis and thus we are confident that the device provided similar magnitudes of assistance across participants. Second, some of our results are limited by the small number of motor units identified. For example, we were unable to extend the muscle-specific results to the GL owing to a lack of motor units identified for this muscle. Although some studies suggest that the GM and GL have similar roles in postural balance [11,13], we would expect different neuromuscular behaviours to stabilize the ankle during balance, as these two muscles produce opposite moments in the ankle's frontal plane [18]. The motor unit results were also limited by the relatively small number of motor units tracked across different conditions, especially for the SOL. Future studies could employ similar methodologies during tasks associated with greater triceps surae muscle activation or use recent developments in high-density EMG electrode design to increase the electrode density [51], thereby maximizing the potential number of motor units identified. Finally, this study purposefully focused on static balance because the decomposition procedures for identifying motor units using high-density EMG are well suited to isometric or static tasks with limited movement of the muscle fibres with respect to the electrodes. In the future, improvements in high-density EMG decomposition algorithms could make it possible to determine whether the individual muscles within the triceps surae exhibit distinct neuromuscular behaviours during dynamic or externally perturbed balance.

In conclusion, our results demonstrated that passive ankle assistance can reduce the muscle activity and the neural drive of the largest plantarflexor muscle while preserving balance performance. The spring-loaded device used in this study primarily offloaded the SOL, which has a more tonic postural activity compared with the GM, GL and TA, which have more phasic activity and were not affected by assistance. The muscle-specific adaptation to the assistive device should be considered when optimizing the design of an exoskeleton or the control of robotic assistive devices based on neurophysiological inputs.

## Supplementary Material



## Data Availability

All data can be found in a repository online at https://doi.org/10.48610/ef34ef9. Supplementary material is available online [52].
