# Supplementary material for: Passive ankle exoskeleton assistance selectively modulates triceps surae neural control during balance
Source: J R Soc Interface. 2026 Oct 14;23(243):20251305. doi: 10.1098/rsif.2025.1305 (PMC13682571; doi:10.1098/rsif.2025.1305)
Supplement: Supplementary file 1 [file rsif.2025.1305.zip › rsif.2025.1305/rsif20251305_si_001.pdf]

Supplemental data to this article can be found online at https://doi.org/10.48610/ef34ef9.
